# Quantification of the Immune Content in Neuroblastoma: Deep Learning and Topological Data Analysis in Digital Pathology

**DOI:** 10.3390/ijms22168804

**Published:** 2021-08-16

**Authors:** Nicole Bussola, Bruno Papa, Ombretta Melaiu, Aurora Castellano, Doriana Fruci, Giuseppe Jurman

**Affiliations:** 1 Data Science for Health, Fondazione Bruno Kessler, 38123 Trento, Italy; bussola@fbk.eu (N.B.); bpapa@fbk.eu (B.P.); 2 CIBIO Department, University of Trento, 38123 Trento, Italy; 3 Department of Paediatric Haematology/Oncology and of Cell and Gene Therapy, Ospedale Pediatrico Bambino Gesù IRCCS, 00146 Rome, Italy; ombretta.melaiu@opbg.net (O.M.); aurora.castellano@opbg.net (A.C.); doriana.fruci@opbg.net (D.F.)

**Keywords:** neuroblastoma, digital pathology, classification, deep learning, topological data analysis

## Abstract

We introduce here a novel machine learning (ML) framework to address the issue of the quantitative assessment of the immune content in neuroblastoma (NB) specimens. First, the EUNet, a U-Net with an EfficientNet encoder, is trained to detect lymphocytes on tissue digital slides stained with the CD3 T-cell marker. The training set consists of 3782 images extracted from an original collection of 54 whole slide images (WSIs), manually annotated for a total of 73,751 lymphocytes. Resampling strategies, data augmentation, and transfer learning approaches are adopted to warrant reproducibility and to reduce the risk of overfitting and selection bias. Topological data analysis (TDA) is then used to define activation maps from different layers of the neural network at different stages of the training process, described by persistence diagrams (PD) and Betti curves. TDA is further integrated with the uniform manifold approximation and projection (UMAP) dimensionality reduction and the hierarchical density-based spatial clustering of applications with noise (HDBSCAN) algorithm for clustering, by the deep features, the relevant subgroups and structures, across different levels of the neural network. Finally, the recent TwoNN approach is leveraged to study the variation of the intrinsic dimensionality of the U-Net model. As the main task, the proposed pipeline is employed to evaluate the density of lymphocytes over the whole tissue area of the WSIs. The model achieves good results with mean absolute error 3.1 on test set, showing significant agreement between densities estimated by our EUNet model and by trained pathologists, thus indicating the potentialities of a promising new strategy in the quantification of the immune content in NB specimens. Moreover, the UMAP algorithm unveiled interesting patterns compatible with pathological characteristics, also highlighting novel insights into the dynamics of the intrinsic dataset dimensionality at different stages of the training process. All the experiments were run on the Microsoft Azure cloud platform.

## 1. Introduction

Neuroblastoma (NB) is the most common cancer diagnosed in the first year of life [1], affecting the sympathetic nervous system. NB is a heterogeneous disease with different outcomes, ranging from spontaneous regression to aggressive progression, metastasis and death. Two main staging systems have been created to stratify patients based on the wide range of outcome and tumor biology: the International Neuroblastoma Staging System (INSS), introduced in 1988 [2] and revised in 1993 [3], and the International Neuroblastoma Risk Group Staging System (INRGSS), introduced in 2009 by the International Neuroblastoma Risk Group task force [4]. INRGSS enhances INSS by defining a series of imaging defined risk factors based on radiological data, such as CT scans and MRI, assessing whether the tumor is circumscribed, if it has metastasized or if it develops near vital parts of the body. However, the effort of establishing an accurate staging system is still ongoing: for instance, additional factors, like the *MYCN* status, the histopathologic classification, and the DNA content have also proven to be significant [5], and they are currently evaluated in clinical practice. Tumor microenvironment (TME) has been shown to play a role in tumor development. In particular, several pieces of evidence highlighted the importance of the composition, density and distribution of tumor-infiltrating lymphocytes as prognostic markers in several human cancers, including NB. Such observations are stimulating a growing research flow, targeting the dynamics of the immune system during the NB evolution [6], following the Precision Medicine paradigm [7,8,9,10].

**Figure 1:**
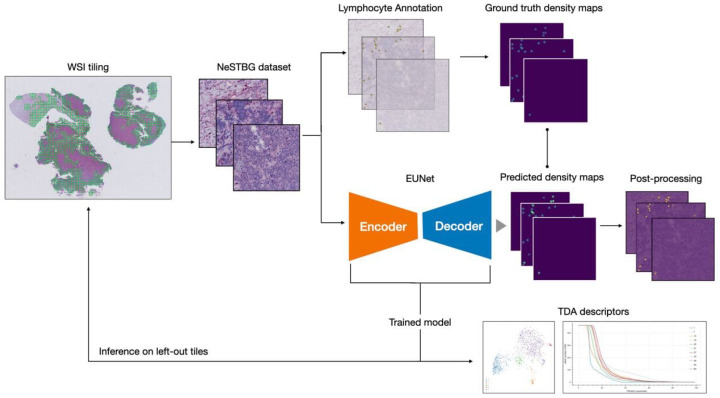
Graphical representation of the full analysis workflow. From the original WSIs, the collection of tiles constituting the NeSTBG dataset is generated and annotated to obtain the ground truth for the model (Section 4.1). Tiles are then used as the input for the DL architecture EUNet (Section 4.2 and Section 4.3) to predict density maps (Section 4.4), that are then post-processed and analysed via TDA descriptors to interpret the detected deep features (Section 4.5).

Here, we propose a novel artificial intelligence (AI) procedure, sketched in Figure 1, for the quantitative evaluation of the immune content in NB specimens in an immunohistochemistry (IHC) / digital pathology (DP) framework, i.e., using whole slide histopathological images (WSIs) as input data: in detail, a deep learning (DL) predictive model is trained to estimate the density of lymphocytes over the whole tissue area of the WSIs. The approach is demonstrated on the neuroblastoma specimens with T-Lymphocytes - Bambino Gesù (NeSTBG), an original dataset of samples from NB patients, provided by Ospedale Pediatrico Bambino Gesù (OPBG) in Rome, achieving a satisfying performance (MAE≈3.1). To boost the reproducibility and interpretability of the DL model, the extracted deep features are analyzed by topological data analysis (TDA) methods [11,12] and, in particular, persistent homology (PH) [13]. To date, this is the first realization of an explainable artificial intelligence (XAI) reproducible platform, encompassing all the analysis steps, from WSI preprocessing to clinical feature interpretation, integrating topological concepts with deep architectures. Although currently more an effective proof of concepts than a fully fledged infrastructure, the novel link established between DL and TDA in DP can lead to further developments along this research line.

## 2. Related Works

### 2.1. Immunohistochemistry

The immunohistochemistry (IHC) technique is particularly suitable to evaluate the density of tumor infiltrating immune cells on histology specimens [14]; by employing the right binding antibody, it is indeed possible to highlight specific immune cells on the tissue, allowing pathologists to obtain information about their localization within the tumor microenvironment. Given the importance of the tumor infiltrating lymphocytes (TILs) in recognizing and neutralizing cancer cells, several studies have been conducted on different tumor types [15,16,17], and the key prognostic significance of these cells has been highlighted. In NB, the study of the immune response can be traced back to more than 50 years ago [18,19,20,21]. However, the adoption of IHC to evaluate the role of the immune infiltration for the prognosis of NB patients has its landmark in the work by Mina and colleagues [22]. The authors have demonstrated that tumor-infiltrating T cells have a prognostic value greater than, and independent of, the criteria currently used to stage NB. In this thorough study, different IHC biomarkers were used, including the Cluster of Differentiation 3 (CD3). CD3 is a complex of trans-membrane proteins, representing an appropriate target for T Cells, also used as a representative marker in the present work. As a major result of [22], a positive correlation is found between the density of CD3 positive cells (i.e., CD3+ cells) and the overall patient survival.

### 2.2. Digital Pathology and Artificial Intelligence

The term DP entails the manipulation of pathology information in a digital environment [23]. In particular, DP studies have digitised tissue glass slides, typically retrieved at a resolution of 20× or 40× [24]. While a high magnification is important to study relevant structures in the tissue, it also represents a technical difficulty: a biopsy specimen scanned with magnification factor 40× has a resolution of ∼0.25 µm/pixel and a color depth of 24 bits. Therefore, approximately 48 MB are needed to represent only 1 mm${}^{2}$ of tissue. As the typical glass slide is much bigger (around 25 mm${}^{2}$), the corresponding WSI file is a $10^{5}\times 10^{5}$ RGB images (called Gigapixel), which typically exceeds the GB [24], making the time required for a single human analysis almost prohibitive. Furthermore, even though many compression techniques exist, their adoption is generally not advised, due to the potential introduction of artifacts hiding possibly interesting patterns.

Similarly to what happened to several other sectors, the novel DL paradigm has revolutionized DP, leading to a fast growing flow of publications for a wide range of pathologies [25,26,27,28,29,30]. For example, Nagpal et al. [31] develop and validate a DL based system for automatic Gleason grading of prostate cancer, while in [32], the authors use artificial neural networks (ANNs) on WSIs to predict the survival of patients in a pan-cancer study. Although WSI labels usually refer to the whole slide, given their high complexity, predictions are typically performed at the level of small patches (aka tiles), extracted from the original WSI: this procedure is known as the weak labelling problem. An interesting approach to solve the weak labelling problem is used in [33], where a meaningful WSI compression is proposed to subsequently train a CNN on the whole compressed image. A similar strategy is also adopted in [34], with the further addition of an attention mechanism. Working with tiles, however, requires careful planning of the model training, not to incur in unwanted biases such as the data (or information) leakage: whenever tiles are extracted from the same WSI in both the training and the validation set, model results are heavily affected by overfitting [35].

### 2.3. Lymphocyte Detection and Density Maps

As pointed out in several references [36,37], detecting and quantifying lymphocytes represent a powerful tool to identify strong prognostic and predictive biomarkers for evaluating cancer progression and targeting novel therapeutic solutions. Nonetheless, it is widely acknowledged that the technical challenges to be solved towards the goal are numerous and difficult, making the aforementioned tasks very difficult to tackle and indeed still an open problem. No shared consensus has been reached by the community on an optimal methodology: automatic localization and quantification of lymphocytes have represented a major goal in DP in the last decade, resulting in a constant stream of publications, featuring the emerging solutions in both imaging and learning, together with dedicated reviews detailing such evolution [38]. Focusing on the methods adopting DL algorithms, convolution is the natural tool common to many proposals, starting from [39], where CNNs were combined with a probability map to identify lymphocytes’ centers. Other approaches have employed CNNs as a classifier to discriminate lymphocytes from the image background [40], generating a heatmap, representing the probability of each pixel being a lymphocyte. The strategy to move from the heatmap to the lymphocytes identification was later improved in [41] through thresholding, while clinical relevance was made explicit by detecting local spatial features [42]. Further technical improvements on the same directions were achieved in [43] with the development of a non-maxima suppression (NMS) algorithm to locate the center of each lymphocyte. Finally, the combination with a more classical morphologically oriented procedure [44] allowed Li and coauthors [45] to better identify the center of each candidate cell.

The landscape of solutions is quite rich, even when restricted to the detection of lymphocytes in IHC-stained WSIs. A first approach combining CNNs with color deconvolution to produce a probabilistic heatmap [46] was later improved in [47] again via an NMS procedure. An important breakthrough came when the general YOLO architecture [48] was adapted to the lymphocyte detection task. After the first attempt [49], where a non-maxima suppression algorithm was used during inference to consider overlapping bounding boxes as detecting the same lymphocyte, in 2019, Van Rijthoven and colleagues proposed YOLLO [50], a modified version of YOLO [48], as a fast method to detect lymphocytes in IHC-stained WSIs. The proposed modifications to YOLO included a guided sampling strategy and a simplified architecture, resulting in both a performance gain and a procedure speed-up. Finally, in [37], YOLLO combined with non-maxima suppression is compared to other approaches based on U-Net, a fully convolutional neural network, and locality sensitive method (LSM).

More recently, although alternative approaches are actively pursued [51,52,53,54], well consolidated methodologies derived by CNNs are still being used [55]. In particular, two families of algorithms deserve a mention for the rather large popularity gained in the last few years, both stemming from the original R-CNN model [56]. The former set of architectures is mainly aimed at quick object detection, with fast R-CNN [57] as the first implementation, followed by its improved version, faster R-CNN [58]. These models still work as building blocks for recent solutions in DP, as in [59,60,61]. The latter family of models stem from the prototypal structure mask R-CNN [62,63], obtained by optimizing the faster R-CNN for pixel-level segmentation tasks. Use of mask R-CNN and derived models is also currently quite widespread in the DP community, with several examples published in the literature [64,65,66,67].

At the same time, crowd counting has always been a challenging task in computer vision. The idea of tackling the counting problem with density maps began with [68]. Then, Zhang and colleagues [69] started using DL models to predict object density maps, later refined in [70] through a new encoder-decoder CNN for crowd counting in aerial images. Similar strategies have been recently used in computational biology for yeast cells detection [71] and in DP [72], where density maps are used to count cells in histology images of bone marrow tissues.

### 2.4. Topological Data Analysis

Topological data analysis (TDA) is a recent approach to data analysis, relying on concepts from algebraic topology [11,73], providing solid qualitative and often also quantitative information about the geometric structure of the considered dataset. In particular, TDA allows the description of topological properties of data as point clouds, time series analysis [74], images [75] or even volumetric and time varying data [76]. From a computational point of view, a great effort has recently been put into building efficient TDA algorithms, data structures and software libraries, such as Ripser [77], Mapper [12], and Giotto-TDA [78]). The grounded theoretical framework and the performing implementations make TDA a powerful data science tool, effectively used nowadays by several labs worldwide for a wide range of applications: a non-exhaustive list of recent examples in bioinformatics is [79,80,81,82,83].

**Figure 2:**
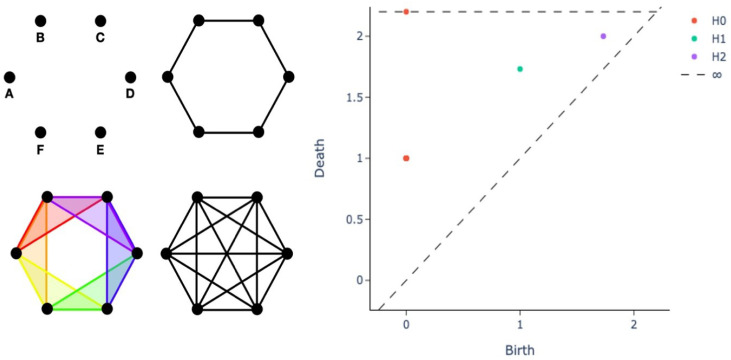
Persistence diagram (**right panel**) for different vietoris rips complexes on equilateral hexagons with side length of 1 (**left panel**). In the left panel, we display the four different categories of vietoris rips complexes generated by 6 points, forming the vertices of a regular hexagon of side length 1 in the Euclidean plane: 0-simplices (**top left**), 0- and 1-simplices (**top right**), 0-, 1- and 2-simplices (**bottom left**) and complexes including simplices of degree higher than 2 (**bottom right**). In the right panel, each point in the scatterplot represents a specific topological feature of the dataset, where the axes denote the values of the distance for which topological features appear (“birth” on the x axis) and vanish (“death” on the y axis).

A fundamental building block of TDA is persistent homology (PH), the geometric technique for studying a system at different length scales and discerning noise from actual topological features, based on the notion of how persistent a feature is throughout all the possible length scales. A compact representation to efficiently encode PH information is offered by the persistence diagram (PD), a visual intuitive way to assess the properties of a dataset and to simultaneously obtain a collection of informative features to be used as input for learning pipelines, supporting the critical step of model interpretability and explainability. Hereafter, we briefly outline the construction of a PD, whose starting point is the geometric concept of a simplex. Consider a finite set of points $S=\{x_{0},\cdots ,x_{n}\}$ that are in general position with respect to the universe $R^{d}$, i.e., *S* should not be contained in an affine subspace of $R^{d}$. If this condition is satisfied, *S* can be associated to a simplex σ(S), the convex hull of *S*. Define the diameter of a simplex as the maximum distance between any two points on the simplex itself, or equivalently, between any of the two vertices, since the set is convex. Given a set of points *S* with diameter *r*, we can define the vietoris rips complex as the set of simplices with diameter d≤r. Moreover, given a vietoris rips complex, it is possible to compute its Betti numbers, where the *k*-th Betti number is denoted as $\beta_{k}\left(X\right)$, for a simplicial complex *X*; in layman’s terms, $\beta_{k}\left(X\right)$ represents the number of *k*-dimensional holes on *S*. For example, consider the vietoris rips complexes shown in the left panel of Figure 2 (adapted from Figure 5.2 in [84]) for different values of *r*.

The four different complexes can be described by Betti numbers as follows:

- The first complex (r=0) is composed of 0-simplices, i.e., the points. Therefore, $\beta_{0}=6$ and $\beta_{k}=0,\;\forall k>0$. Note that $\beta_{0}$ indicates the number of connected components.
- The second complex (r=1) includes 6 0-simplices and 6 1-simplices, denoted by dots and lines, respectively. Here $\beta_{0}=1$ and $\beta_{1}=1$ as there is one connected component and one 1-dimensional hole, namely the circle originated by the connection of the points.
- In the third step we have six 0-dimensional simplices, six 1-dimensional simplices, and six 2-dimensional simplices. The 2-dimensional simplices are the triangles, that is, the connection of 3 points. Thus $\beta_{0}=1$ and $\beta_{1}=1$.
- The last complex (r=2) has simplices of higher degree greater than 2. Here $\beta_{0}=1$ but $\beta_{0}=0$: for this choice of *r* the 1-dimensional hole is filled.

The example in Figure 2 illustrates that features of points arranged, for instance, in a circular shape can be recovered from their topological descriptors. In particular, $\beta_{1}=1$ for a large range of possible distance values: this is thus defined as a persistent feature of the dataset {A,B,C,D, E,F}. PD provides a compact representation of the topological insights provided by Betti numbers, as shown in the right panel of Figure 2.

Betti numbers can be encoded into a two-dimensional scatterplot, each point representing a specific topological feature of the dataset. The *x* and *y* coordinates denote the values of the distance for which topological features appear (“birth” *x*) and vanish (“death” *y*), respectively. Considering (x,y) as coordinates of the scatterplot, only half of the plot is relevant and, the closer a point is to the diagonal, the shorter its lifetime, and thus the point may represent topological noise. The *k*-th Betti number $\beta_{k}$ is the rank of the *k*-th homology group $H_{k}$ and thus each feature counted by $\beta_{k}$ belongs to $H_{k}$. Considering now the plot in Figure 2:

- The point at coordinates (0,1) represents 6 overlapping points. The 6 connected components (points) appear at r=0 and vanish at r=1, the side length of the equilateral hexagon, when each point is connected to its neighbors by a line.
- There is an H1 point (a 1-dimensional hole) with the same birth value of the death of the 6 connected components (r=1), as this topological feature arises from the union of the 6 features.
- A H0 point (a 0-dimensional hole) lies at *∞*; indeed, the connected components represented by the union of the 6 points persist for every value of *r*: for every value of r>1, there exists only one connected component.

Barcodes represent a different visualization of PD, but encode the same information. If a PD is a scatterplot with coordinates given by the length scale for which topological features arise or vanish, a barcode can be regarded as a dumbell plot where each bar represents a different topological feature, and the start and end values of the bar represent its life span. Since both PDs and barcodes are difficult to handle in a ML framework, recently the novel concept of Persistence Landscape was introduced [85] to translate PDs into standard vector spaces by means of piecewise linear functions.

**Figure 3:**
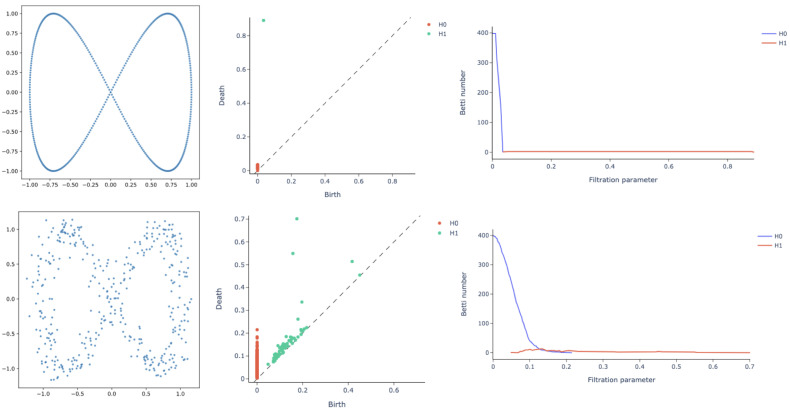
Topological descriptors on a lemniscate-shaped synthetic dataset with (**top**) and without (**bottom**) noise: dataset scatterplot (**left column**), persistence diagram (**central column**) and Betti curves for homology groups H0 and H1 (**right column**). Although it is still possible to appreciate the same topological structure, the persistence diagram for the noisy lemniscate has a cluster of points near the diagonal, representing non-persistent features of the input point cloud. The different spatial organization of the two structures is also reflected by the Betti curve for H0, displaying a slower decay rate.

Finally, Betti curves represent the magnitude of an homology group at different length scales of the filtration. Betti curves are an intuitive way to visualize the evolution of topological features within the dataset. Take, as an example, the equilateral hexagon and its persistence in Figure 2. Recall that, in the persistence diagram, there is only one point at (0,1), which is the collapse of original connected components. By using a Betti curve, it is possible to visualize the number of elements belonging to a homology group at every length scale. In this way, we could have easily observed the Betti curve starting at 6 and decreasing to 1 for r=1. A less trivial example is reported in Figure 3. On the top row, a point cloud with the shape of a lemniscate is created without noise, i.e., the points are equally spaced. In the top row are also illustrated the persistence diagram and the Betti curves for homology groups H0 and H1. Similarly, the bottom row contains a lemniscate-shaped point cloud with a corresponding persistence diagram and Betti curves for H0 and H1, but the point cloud construction involves some noise. The bottom row shows that it is still possible to appreciate the same topological structure, but the persistence diagram is more crowded with points near the diagonal, representing noisy features and thus not persistent features of the input point cloud. The different spatial organization of the two point clouds is also reflected by the H0 Betti curve; for the noisy dataset, it indeed has a slower decay rate.

### 2.5. Umap

Uniform manifold approximation and projection (UMAP) is a novel dimensionality reduction technique introduced in 2018 by McInnes and colleagues [86,87], with roots in the fields of algebraic topology and Riemannian geometry. UMAP is a manifold learning algorithm projecting high-dimensional data in lower spaces. The underlying hypothesis is that data lie on one or more manifolds, whose structure UMAP tries to approximate. In detail, UMAP exploits fuzzy simplicial sets in order to create a topological representation of the manifold. The higher dimensional representation of the manifold is then adapted to the target lower dimensional space via optimization techniques. In this adaptation, the exact points coordinates lose their spatial meaning but points that are close together are more similar than points far apart. In the high dimensional point cloud, UMAP constructs the Čech complex ${\overset{ˇ}{C}}_{\epsilon }\left(X\right)$. Čech complex is the nerve of the set of balls centered on each points and having radius ϵ. By the nerve theorem [88], from ${\overset{ˇ}{C}}_{\epsilon }\left(X\right)$ we can thus recover all the key topological structures of the original data. Notice that the UMAP implementation constructs the vietoris rips complex ${\mathrm{VR}}_{\epsilon }\left(X\right)$ (being ${\overset{ˇ}{C}}_{\epsilon }\left(X\right)\subset {\mathrm{VR}}_{\epsilon }\left(X\right)\subset {\overset{ˇ}{C}}_{2\epsilon }\left(X\right)$), which is computationally easier. UMAP has a strong theoretical foundation as a manifold learning technique and it is faster than many alternative dimensionality reduction algorithms, allowing users to work with large or very high dimensional datasets without requiring excessive computational power. The ability to vary the embedding dimensionality allows UMAP to be used for more than just data visualization: for instance clustering, when coupled with the HDBSCAN algorithm. UMAP has also been adopted to investigate ANNs. One example is the activation atlas by Carter and colleagues [89], using UMAP to explore the distribution of activation maps from hidden layers of an Inception V1 network [90], enlightening how different filters of the artificial neural network are correlated. Another example is [91], where UMAP loss is extended to DL thus improving classifier performance by better capturing data structure. Nonetheless, initialisation seems to be critical and deserves special care [92].

### 2.6. Hdbscan

**Figure 4:**
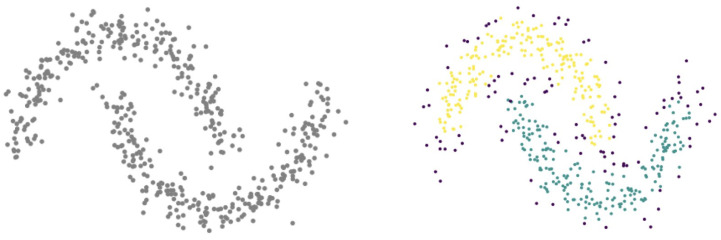
HDBSCAN performance (**right**) on double crescent shaped clusters (**left**) in $R^{2}$.

Hierarchical density-based spatial clustering of applications with noise (HDBSCAN) [93] is an extension of the classic DBSCAN algorithm [94], improved by providing a hierarchical structure of clusters found from density estimation and a more intuitive approach for cluster selection. The density-based approach can identify clusters with arbitrary shapes, thus overcoming limitations of algorithms that are able to work only with convex clusters such as K-means. An example of HDBSCAN applied to arbitrarily shaped clusters in $R^{2}$ is shown in Figure 4.

The main advantage of HDBSCAN relies on the simplicity of tuning its key hyperparameters, namely the minimum cluster size, and the number of neighbors used to estimated the density for each point in the dataset. The hierarchical, density based approach is also robust with respect to subsampling. Furthermore, the HDBSCAN algorithm can count on really fast implementations [95].

### 2.7. Twonn

In a first attempt to understand deep features, Odena and coworkers [96] used deconvolution layers to explore the filters learned by a CNN, while few years later Carter and colleagues [89] used UMAP to explore activation maps coming from different layers of an Inception network. More recently, the Mapper algorithm has been used in [97] to study the structure of CNN filters, while Ansuini and colleagues [98] employed TwoNN [99] to estimate the intrinsic dimensionality of a dataset and how such dimension changes when the dataset is transformed by the different CNN layers. TwoNN is a recent method that can be used for the estimation of the intrinsic dimensionality of high-dimensional and sparse data [99]. TwoNN assumes that the density of points is approximately constant on the length scale of the distance between a point and its two neighbors. With the former hypothesis, TwoNN uses information only from a restricted neighborhood of the point to measure properties of the data manifold [98]. The quantity $\rho_{i}=\frac{d_{i,2}}{d_{i,1}}$ is assumed to be a random variable following a Pareto distribution; if points are uniformly sampled and the hypothesized manifold has intrinsic dimensionality d∈[0,+∞], then $p\left(\rho_{i};d\right)=d\rho_{i}^{-(d+1)}$. From this formula, the parameter *d* can be estimated by fitting the likelihood function to the data $P\left(d;\rho_{i}\right)=d\rho_{i}^{-(d+1)}$, where $\rho_{i}$ is known.

## 3. Results and Discussion

### 3.1. Quantification of the Immune Content

To quantify the immune content in NB in terms of lymphocyte detection, a suite of DL experiments were run on the NeSTBG dataset, employing a U-Net network with an EfficientNet-b3 architecture as encoder (EUNet for short). The whole dataset was first partitioned into training (TR) and test (TS) subsets, with ratio 34−14; on the TR portion, a 5-fold cross validation was run four times (TR-CV), and the model trained on the whole TR was then evaluated on the left-out TS. The outcome of the prediction on TS was finally postprocessed (TSp) to enhance the lymphocyte detection: for this model, Precision = 0.73, Recall = 0.85 and F1−score = 0.75. The complete set of classification performance is summarized in Table 1.

**Figure 5:**
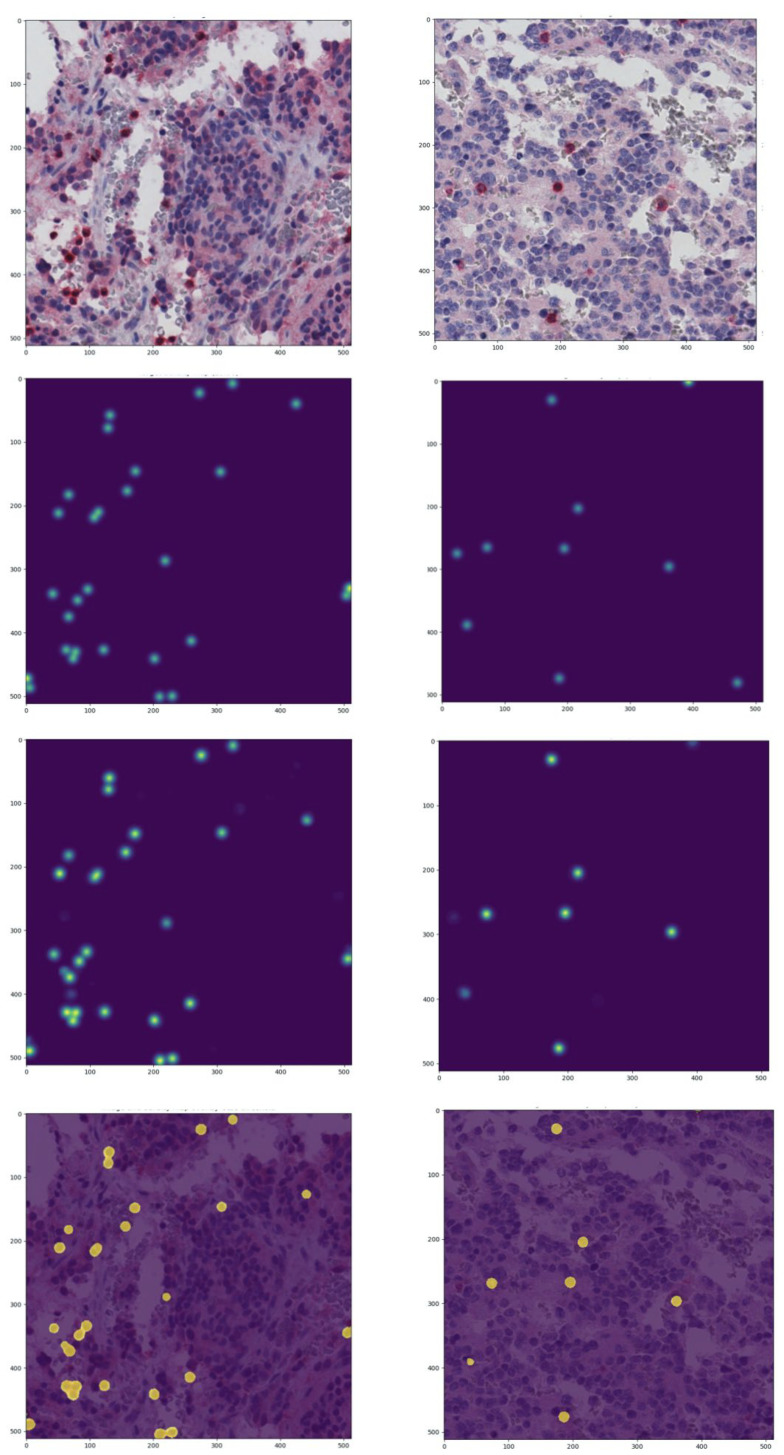
Examples of the density map prediction process on two tiles. **First row**: original images. **Second row**: ground truth density maps. **Third row**: predicted density maps. **Fourth row**: predicted density maps discretized with Otsu threshold overlaid on the original image.

**Figure 6:**
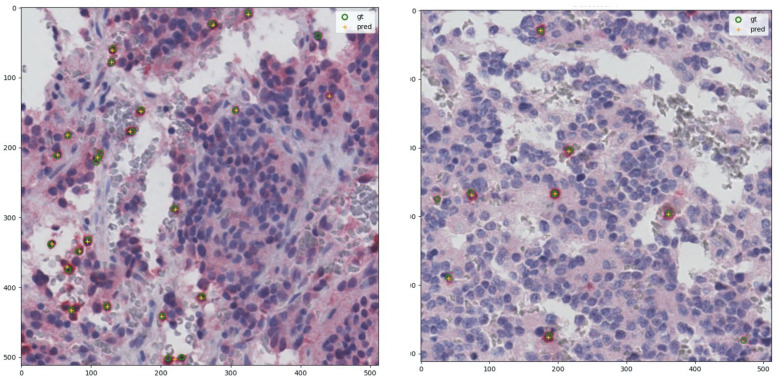
Examples of lymphocytes detection obtained through postprocessing of predicted density maps on the original tiles of Figure 5. gt: ground truth after postprocessing, pred: prediction.

The EUNet was later applied to the entire tissue area of the 54 NeSTBG WSIs to obtain a patient-wise estimate of T-cell density. The tiles already included in NeSTBG were discarded during the training phase to avoid data leakage effects. Note that, for each WSI, NeSTBG includes approximately 1100 of all possible tiles. In Figure 5, the process of density estimation is graphically summarised on two tiles, while in Figure 6, the effect of postprocessing on the same two tiles is shown.

To compute the density, the area (mm${}^{2}$) of a single tile of size 512×512 pixels can be approximated as $A_{\mathrm{tile}}=l^{2}$ = 0.655 mm${}^{2}$ where $l_{\mathrm{tile}}$ = (512 pixelmm ·ρ·$10^{-3}$) = 0.256 mm is the tile side length and ρ = 0.5 µmpixel is the resolution (20×) used for the tile extraction.

**Figure 7:**
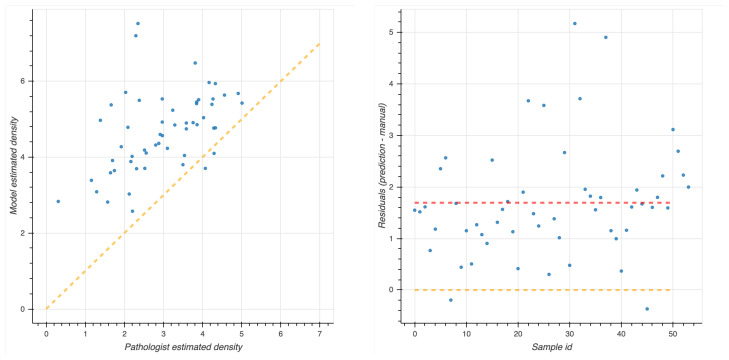
Comparison between DL and human expert density estimations computed for the 54 patients of the NeSTBG dataset. Notice that the pathologist estimation is computed on 10 regions of interest while the DL predicted densities are computed on the whole slide. **Left panel**: correlation plot for predicted densities and density estimated by pathologists. **Right panel**: residual plot for difference between DL and pathologist density estimation. Yellow line, both panels: perfect correlation. Red line: average difference between DL and pathologist prediction.

As a benchmark, the DL estimate is compared with the manual estimate of a pathologist through the formula proposed in the reference work [22], expressing the density estimate *L* for each slide as the natural logarithm of the number of lymphocytes per mm${}^{2}$:

$$L=\log\left(\frac{1}{n}\sum_{i=0}^{n}\frac{c_{i}}{A_{i}}\right)\;,$$

where *n* is the number of regions of interest (5 or 10), log is the natural logarithm, $c_{i}$ is the number of lymphocytes in the *i*-th selected region of interest, and $A_{i}$ is the area of the *i*-th region of interest expressed in mm${}^{2}$. The two density estimates have a Pearson correlation coefficient of 0.47 with *p*-value $3\times 10^{-4}$: in detail, in Figure 7 the corresponding correlation plot is shown, together with the residual plot displaying the difference between DL-predicted density value and pathologist estimation, indicating a positive offset.

### 3.2. Clinical Assessment of the Topological Features

**Figure 8:**
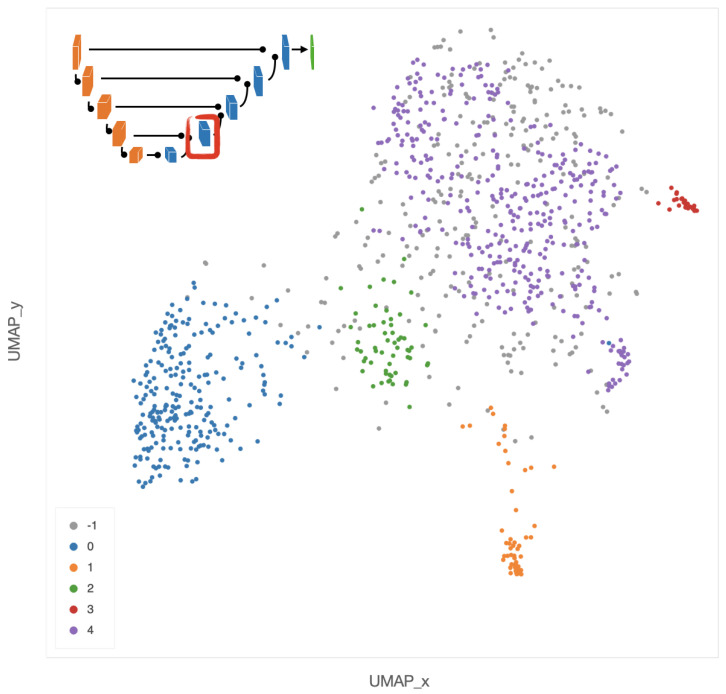
UMAP 2-dimensional embedding of a deep EUNet layer with cosine metric, and HDBSCAN clustering assignments. Gray points (label −1) are classified as noise by the clustering algorithm, while colored points belong to the clusters 0–4 detailed in Figure 9, Figure 10, Figure 11, Figure 12 and Figure 13: stroma rich areas with low TILS level (0), tissue with septa infiltration (1), tissue with pseudo-necrotic tissue infiltration (2), intermediate level of lymphocyte infiltration in stroma poor areas (3) and low level of infiltration in stroma poor areas (4). In the upper-left corner, the graphical schema of the corresponding layer in EUNet.

Clustering analysis was performed by HDBSCAN on the deep features projected by UMAP from the deepest (central) layers of the EUNet. Notably, these features are represented by vectors $v_{i}\in R^{D}$, with dimension *D* = 524,288, as the output of the feature maps in the deepest layers has spatial dimensions 128×128 and 64 feature channels. The most interesting structure emerged in the second block of the EUNet decoder; Figure 8 shows the cluster assignment using cosine similarity as metric in the higher dimensional space, 15 neighbours and zero minimum distance for UMAP, minimum cluster size 5 and minimum number of samples 16 for HDBSCAN.

**Figure 9:**
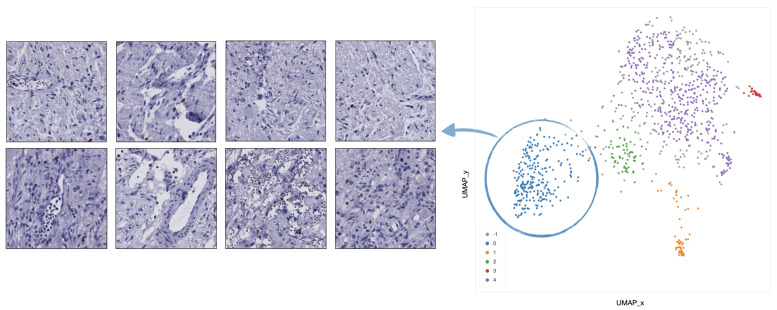
Example of 8 representative tiles with stroma rich areas with low level of TILs, grouped as cluster 0 by UMAP and HDBSCAN.

**Figure 10:**
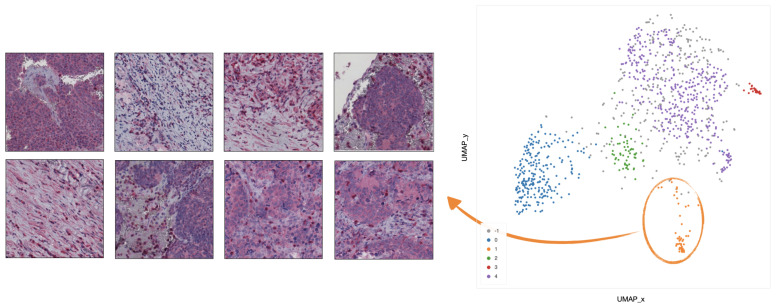
Example of 8 representative tiles with infiltration inside septa, grouped as cluster 1 by UMAP and HDBSCAN.

**Figure 11:**
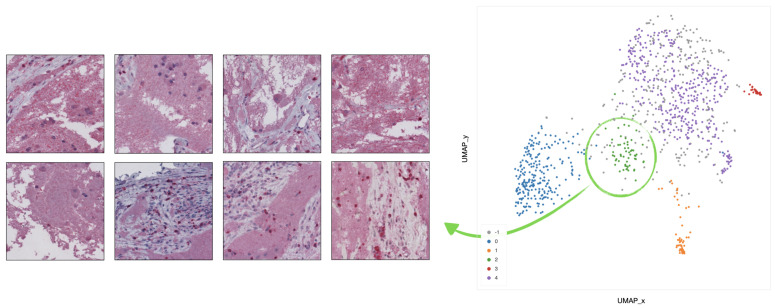
Example of 8 representative tiles with infiltration of lymphocytes in pseudo-necrotic tissue, grouped as cluster 2 by UMAP and HDBSCAN.

**Figure 12:**
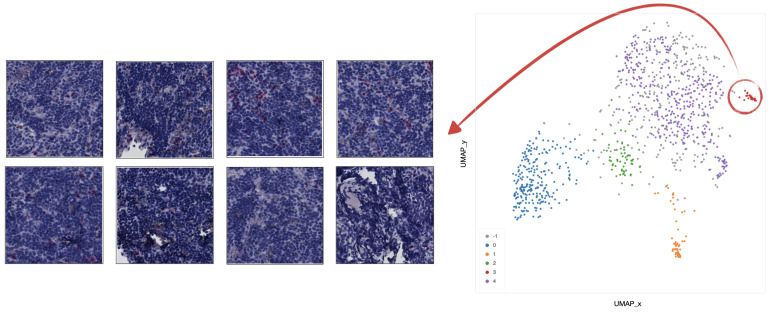
Example of 8 representative tiles with an intermediate level of lymphocyte infiltration in stroma poor areas, grouped as cluster 3 by UMAP and HDBSCAN.

**Figure 13:**
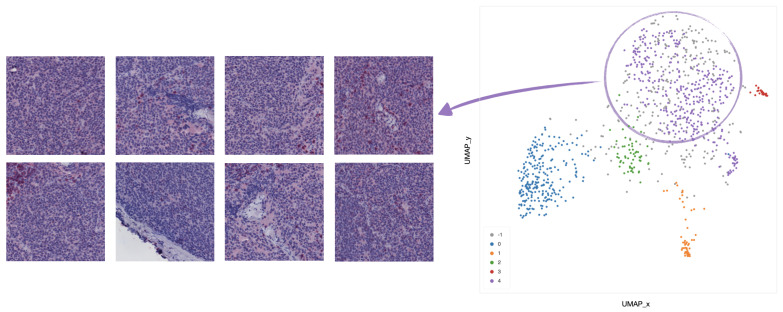
Example of 8 representative tiles with a low level of infiltration in stroma poor areas, grouped as cluster 4 by UMAP and HDBSCAN.

The tiles belonging to the 5 clusters identified by HDBSCAN can be clinically characterized according to their spatial arrangement. In particular,

- In cluster 0 (blue), the majority of tiles represents stroma rich areas with low level of TILs (Figure 9).
- In cluster 1 (orange), the majority of the tiles represents tissue with infiltration inside septa (Figure 10).
- In cluster 2 (green), the corresponding tiles present infiltration of lymphocytes in pseudo-necrotic tissue (Figure 11).
- In cluster 3 (red), the corresponding tiles show an intermediate level of lymphocyte infiltration in stroma poor areas (Figure 12).
- In cluster 4 (purple), the corresponding tiles display a low level of infiltration in stroma poor areas (Figure 13).

**Figure 14:**
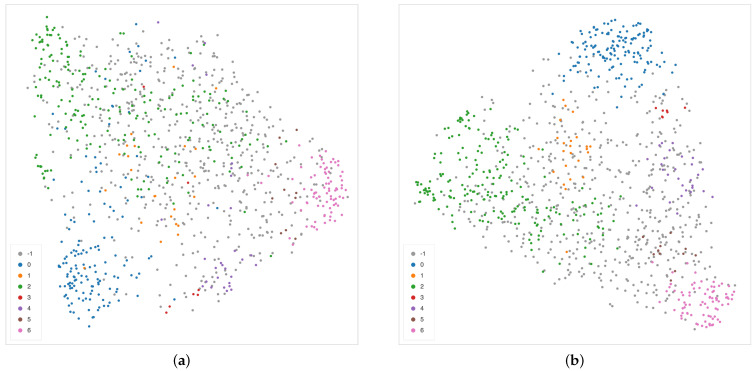
Alternative UMAP 2-dimensional embedding on the features extracted from the second layer of the decoder path of the EUNet network, with $L_{1}$ (**a**) and $L_{2}$ (**b**) metric. Colors correspond to the detected clusters.

The cosine metric seems to be more effective in detecting sub-structures among samples described by DL features than alternative distances such as $L_{1}$ or $L_{2}$, as shown in Figure 14.

Here, sparsity plays a crucial role: data represent activation maps returned by a rectifier linear unit (ReLU) layer [100] inducing sparsity on the data. Indeed, the extracted feature vectors are quite sparse, with about 60% of the entries being zero, on average. Given the high-dimensionality and the sparsity, cosine similarity is more effective than $L_{p}$ alternatives [101].

**Figure 15:**
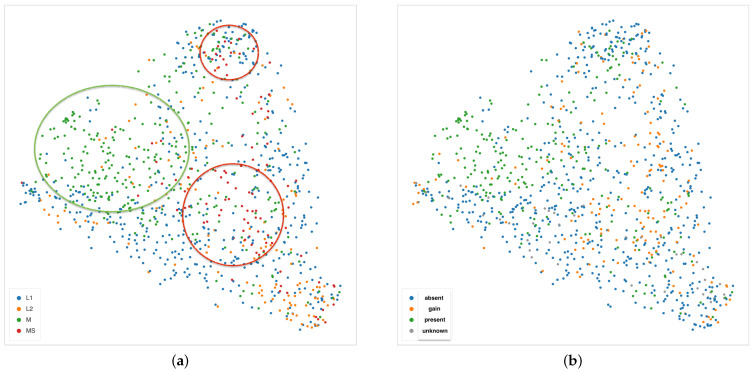
UMAP 2-dimensional embedding on the second layer of the decoder path of the EUNet, with Euclidean metric, minimum distance 0.02 and 15 neighbours. (**a**) Color indicates INRGSS. The red and green ovals mark the plot areas enclosing the majority of NB patients of stage MS and M, respectively. (**b**) Color indicates *MYCN* amplification.

Nonetheless, an interesting pattern emerges also from the UMAP projection of the second layer of the decoder using the Euclidean distance, shown in the two panels of Figure 15. In the left panel, colors represent the INRGS stage of the NB patients, while in the right panel, NB patients are represented according to their *MYCN* amplification status. In the left scatterplot, high-risk NB patients from stage M are mostly localized on the left portion of the point cloud. Tiles from patients in the L1 stage can be mainly found along the sides of the triangular shape and, finally, most of the tiles from MS patients (with metastases but with favorable prognosis) lie in the centers spreading to the upper and bottom-right vertex of the triangle. Notably, patients with *MYCN* amplification are clustered together in the upper-left area of the scatter cloud, similarly to high-risk NB patients.

### 3.3. Topological Analysis of the Deep Features

**Figure 16:**
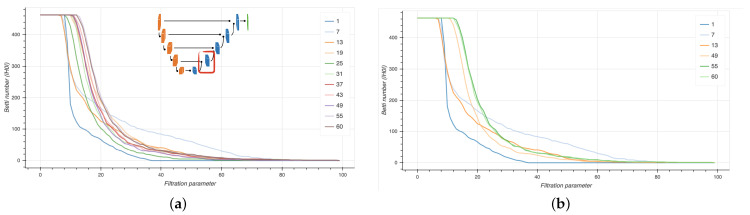
Betti curves for the 0-th homology group H0 from the third decoder block (inset) at different epochs (**a**) and in particular for the first and last three epochs (**b**).

We computed persistence diagrams (PD) to extract Betti curves from six selected EUNet blocks at different stages during model training. In Figure 16, Betti curves are shown for the 0-th homology group H0 from the third decoder block at different epochs (left panel), with a focus on first three and last three epochs (right panel).

Notice that the Betti curves become smoother as the training proceeds, suggesting that the EUNet is progressively learning a meaningful representation of the data. At earlier training stages, several groups of connected components are merged together at uniformly spaced thresholds; later in the process, the curves decrease slower, implying that, from a set of points lying at uniform distances, there are larger groups at non-uniform mutual distances. Finally, towards the end of the training, points become less and less uniformly distributed, as indicated by the smoother profiles of the curves.

### 3.4. Intrinsic Dimensionality of Datasets

**Figure 17:**
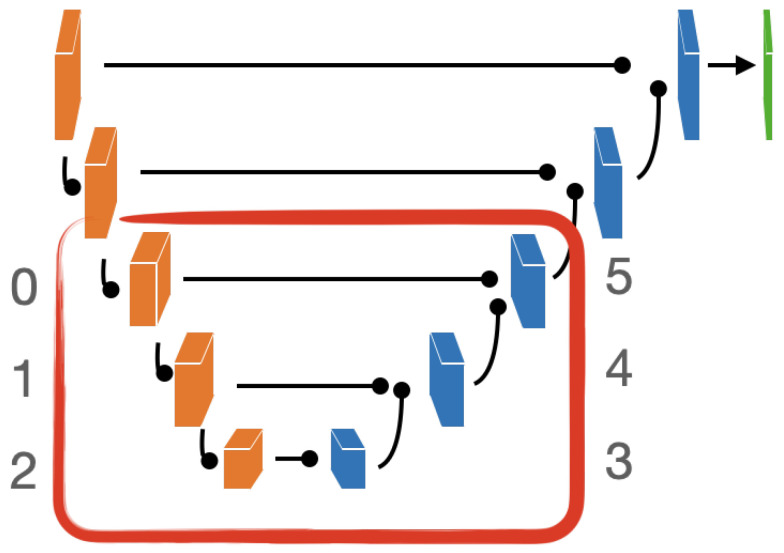
Graphical representation of the EUNet architecture. In the red encircling, the 6 inner blocks computing the Intrinsic Dimensionality at different stages of the training process using the TwoNN algorithm.

The intrinsic dimensionality (ID) of NeSTBG is computed by the TwoNN algorithm in the six inner blocks of the EUNet (Figure 17) at different stages of the training process. Despite the high dimensionality of the deep feature space, the NeSTBG dataset possibly lives on a manifold of much lower dimension, similarly to the findings in [98]. Specifically, we computed the activation map from the EUNet model state every six epochs, and we estimated the dataset ID. Notably, ID=125 for the original dataset (computed on 20 random tiles extracted from each patient), while ID=26 for the predicted density map.

**Figure 18:**
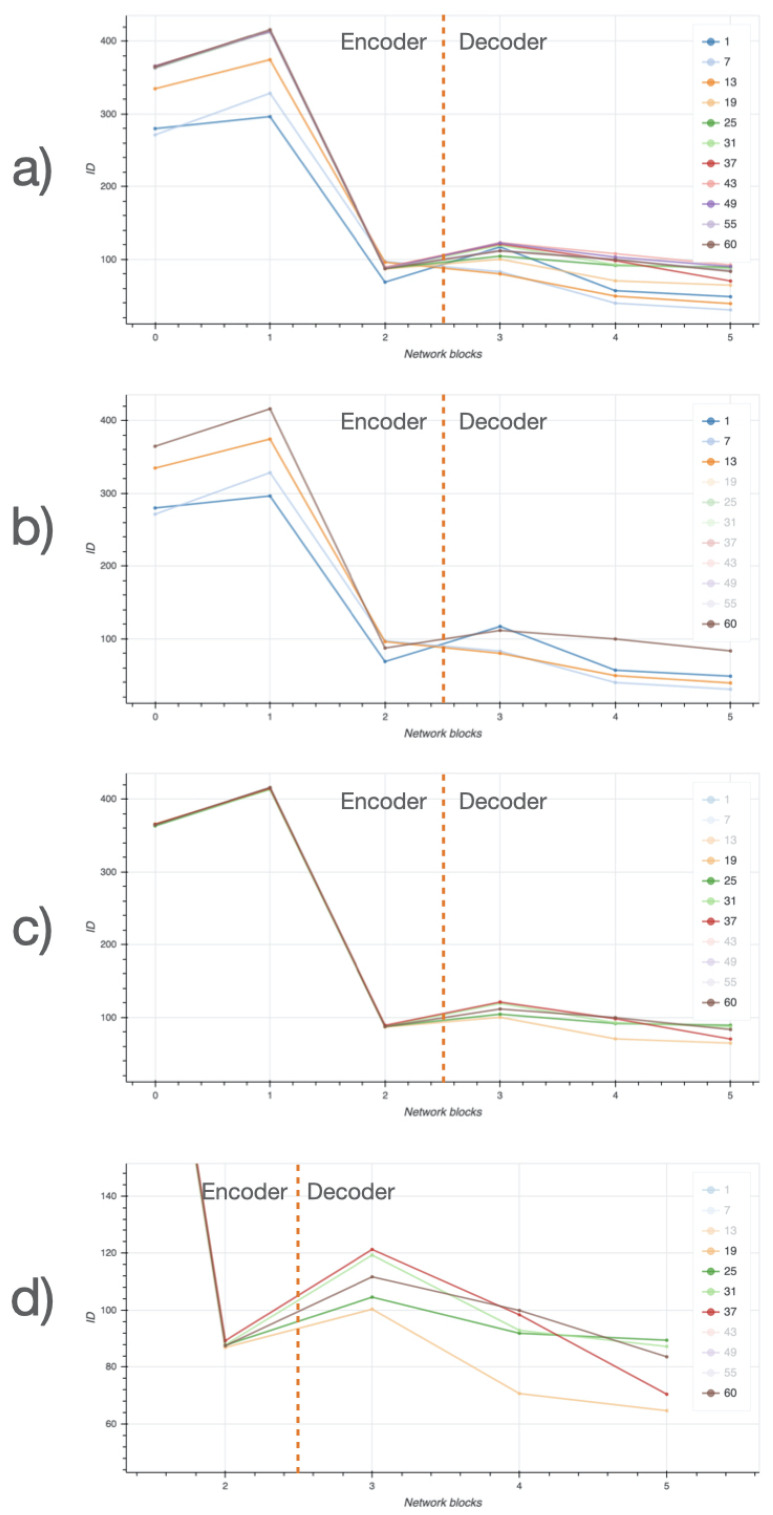
Intrinsic dimensionality (ID) along different layers of the EUNet network, at different training stages. (**a**) ID across layers for all training checkpoints, (**b**) ID across layers for first three training checkpoints, (**c**) ID across layers for intermediate training checkpoints, (**d**) ID across layers for intermediate checkpoints, zoomed on the decoder. Legend includes all different epochs; ID curves corresponding to transparent elements of the legend are not shown.

Detailed dynamics of the ID estimates are reported in Figure 18. In the top panel, the ID is plotted for each inner block for all the training epochs. In (panel b), ID is plotted for the first three epochs (1,7,13) and for the last epoch (60), which corresponds to the highest peak of the encoder. During the central epochs (panel c) ID values of the encoder are stable, while the ID values of the decoder still show some variability; in particular (panel d) a ID peak on the third block. Thus, ID dynamics share a similar trend in both the encoding and the decoding phase, at different magnitudes.

## 4. Materials and Methods

### 4.1. The NeSTBG Dataset

**Figure 19:**
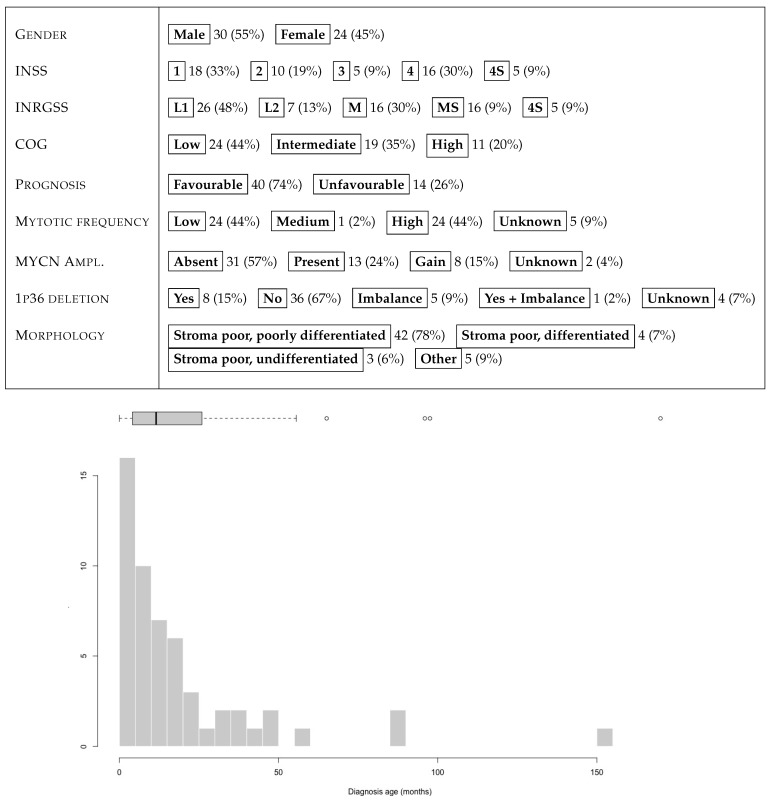
Summary of clinical features and age distribution at diagnosis (month) for the 54 patients of the NeSTBG dataset.

The NeSTBG dataset is a collection of 3782 tiles with annotations for the centers of lymphocytes for 54 IHC-stained WSIs of as many NB specimens, previously characterized for density of tumor infiltrating immune cells, including T cells [22], dendritic cells and natural killer cells [102], as well as the expression of PD-L1 and PD-1 [103]. CD3 stained slides were digitalized by the Menarini D-Sight scanner at native magnification 40× (resolution 0.25 µmpixel ) and employed for digital analysis. The 54 patients are reasonably gender balanced (30 males vs. 24 females), mostly younger than 4 years at diagnosis. INSS, INRGSS and COG values are quite heterogeneous, as well as the tumour location, with suprarenal position as the most frequent (24 patients, 44%); less frequent locations include lymph nodes, aorta, scapula, eye, pharynx, and spleen. The full set of clinical characteristics of the 54 patients are summarised in Figure 19. Morphologically, the large majority of the tumours in the cohort are stroma poor (91%), and in particular poorly differentiated (42 patients, 78%). The remaining 12 samples include 4 differentiated and 3 undifferentiated stroma poor cases, together with an undifferentiated case and 4 ganglioneuroblastoma (GNBL), with only a single stroma rich case. Furthermore, at a 40× magnification level, all samples have about 560 tumoral cells in each sector, while pseudonecrosis areas are mainly present in Stage 4 samples. Note that the heterogeneity of the stroma in the cohort does not represent a confounding factor in the analysis: our experience suggests that immune cells can infiltrate the tumour tissue regardless of the morphology of the stroma, thus yielding that tissue composition is not directly correlated to the immune content. Furthermore, CD3 staining is extremely clean and specific, and the background noise is reduced by precise stain tuning and by blocking the non-specific binding sites, with no need for preprocessing procedures reducing stain variability. Each tile in NeSTBG is a 512 by 512 pixels RGB image stored in *png* format, randomly extracted from a WSI at 20× magnification.

Annotations refer to the *x* and *y* coordinates of the centers of the lymphocytes found in each tile. Level 1 in the OpenSlide standard [104], corresponding to 20× magnification and 0.5 µmpixel resolution, was selected for the images as a trade-off between image details and computational load, being sufficiently detailed to detect marked cells and to describe WSIs using a limited number of tiles. Segmentation of the tissue region within the slide was also needed: a large portion of WSI is background, and restricting computations only on the tissue area saves both time and resources. However, the original slides in the NeSTBG dataset included many types of artifacts, for instance different appearances of the background surrounding the tissue; WSIs presented a wide range of shades, from pure white to greys with different level of details.

**Figure 20:**
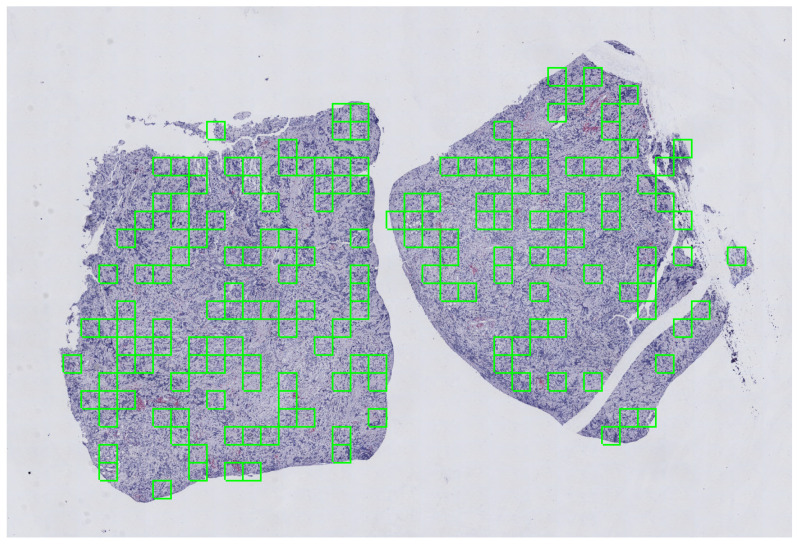
Visualization of the random extraction pattern for the tile extraction in a grid-like fashion. A portion of an CD3+ stained WSI used for the NeSTBG dataset is portrayed (at magnification 1.25×). The size of each tile is representative of the real portion of tissue captured with a 512×512 tile at 20× magnification.

To address the above issues, a sequence of filters were applied to mask out low frequency areas, and morphological operations were used to refine the result. The extraction scheme was designed by overlaying a grid on the tissue area detected on each slide, where each cell of the grid represented a tile. A random number of tiles ranging from 20 to 175 were extracted with random uniform probability, in order to have a representative sample of tissue per slide. The pre-processing steps have been performed with the *histolab* library (https://github.com/histolab/histolab) (accessed on 13 August 2021), a recently introduced open source Python package for reproducible preprocessing in DP. An example of the tile extraction procedure is shown in Figure 20.

**Figure 21:**
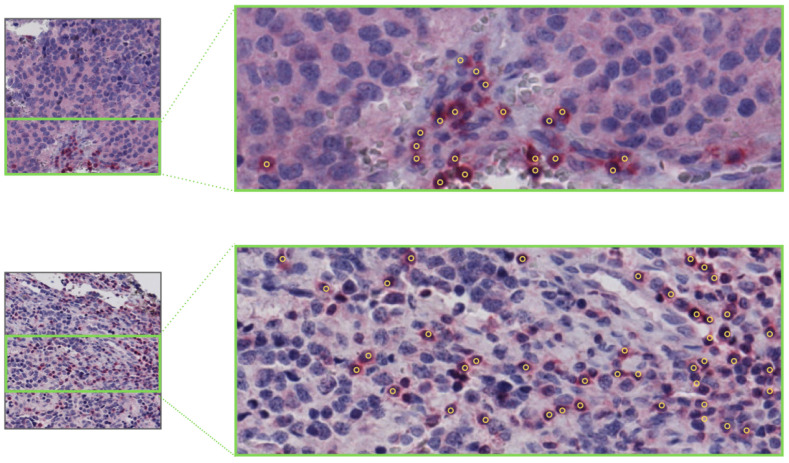
Example tiles from NeSTBG with corresponding manual point-wise annotations for the centers of the lymphocytes by the VIA software.

The point-wise annotations for the centers of the lymphocytes were manually performed using the VIA annotation tool (version 2) [105] by four trained annotators, generating 73,571 annotations for 3782 tiles extracted from a total of 54 WSIs. Examples of annotations are reported in Figure 21.

Given the non-negligible irregularity in the shapes of lymphocytes, the staining variability, and the presence of really packed clusters of T-cells, a relaxed constraint for the annotations was chosen, following the strategy introduced in [68] for object counting in crowded scenes; the authors defined a density map of the objects in a crowded scene by centering at each annotated point a Gaussian curve, and normalizing such that the integral over the whole scene would result in the number of objects. When used for lymphocyte detection, the density map approach associates to the annotated centers the highest confidence of objectness [106], a measure that decreases with radial distance from the center.

The point-like annotations were used to build targets to train the deep learning model to reproduce the density maps instead of bounding boxes typically used in an object detection task. This approach allows the model to encode the confidence of the annotation during the training phase, and also to leverage the surrounding context for the prediction. To define a density map, let T be an RGB tile of shape (N×N×3), and *A* its set of annotations

$$A=\{c_{k}=\left(x_{k},y_{k}\right)|k\in \left[0,n\right],\;x_{k}\in \left[0,N-1\right],\;y_{k}\in \left[0,N-1\right]\;\mathrm{for}\;n\in N,\;n\leq \infty \}$$

The density map is then computed as following:

1. Assign a value *d* to each annotated pixel and define $\hat{M}$ as:
  $$\hat{M}\left(i,j\right)=\left\{\begin{array}{c} d\;\mathrm{if}\;\left(j,i\right)=c_{k}\;for\;k\in \left[0,n\right]\\ 0\;\mathrm{otherwise} \end{array}\right.$$
2. Define a Gaussian kernel
  $$G\left(x,y;\sigma \right)=\frac{1}{2\pi \sigma^{2}}e^{-\frac{x^{2}+y^{2}}{2\sigma^{2}}}$$
  and a squared structuring element GK, with side length l<<N and values given by *G* centered on the midpoint of GK;
3. Convolve $\hat{M}$ with GK to obtain the target density map $M=\hat{M}*GK$.

### 4.2. EUNet Architecture

EUNet, the chosen architecture for the predictive model, is based on the fully convolutional U-Net [107] in its encoder-decoder version. The aim of the encoder is to extract feature maps at different depth; the corresponding decoder blocks will up-sample feature maps from preceding layers and use feature maps of the encoder to refine the spatial information. Specifically, for each layer of the decoder:

1. The feature map from the preceding layer is up-sampled with standard up-sampling operations, without any trainable parameter.
2. The up-sampled feature map is concatenated with the feature map from the symmetric level of the encoder path on the depth dimension (i.e., adding more feature channels).
3. The concatenated feature map is fed to convolution operations to refine the spatial information and reduce the number of feature channels.

In this work, we leveraged the PyTorch [108] U-Net implementation provided by Yakubovskiy in [109], which includes several encoder architectures and provides pretrained ImageNet weights [110,111,112].

**Figure 22:**
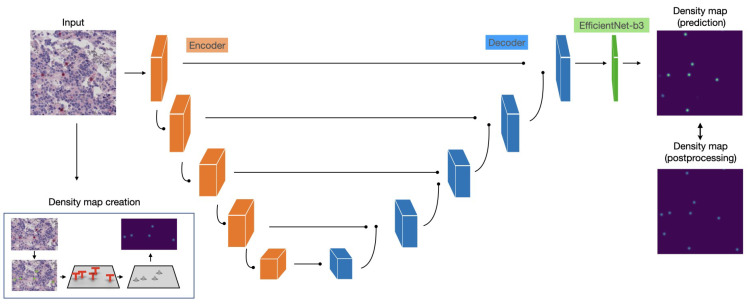
The full EUNet analysis pipeline.

EfficientNet [113] (b3 version) was chosen as encoder; moreover, the spatial and channel squeeze and excitation blocks (scSE) [114] were also introduced in the decoder to improve model performance. The proposed framework is illustrated in Figure 22 and includes:

- encoder and decoder each composed of five blocks;
- scSE blocks at the end of each decoder block;
- Decoder blocks with output feature channels of size: 256, 128, 64, 32, 16;
- Identity function as activation map in the output layer.

The squeeze and excitation blocks (SE), originally introduced in [115], implement a self-attention mechanism to make the network focus on the most relevant feature channels, by first squeezing the spatial dimensions, and then using global information on feature channels to learn a vector of coefficients used as weights for each channel in the input feature map. See Figure 2 in [115] for a graphical scheme of the SE block. In particular, SEs exploit the global average pooling to resize the input feature map $M_{CxHxW}$ to a vector $z_{Cx1x1}$, where *C* is the number of feature channels, *H* and *W* the height and the width, respectively. The vector $z_{C\times 1\times 1}$ is processed through a pipeline including a linear layer that halves its size, a ReLU activation layer, a second linear layer that recovers the original number of channels *C*, and finally a sigmoid activation feeding the vector of weights to the channels of the input feature map. Two different versions of SE were later introduced in [114,116], aimed at improving segmentation models by introducing spatial attention components. The former, named squeeze and excitation, works by first learning a mapping that reduces the number of channels in the input feature map from *C* to 1, hence summarizing information from the *C* input channels to a single number for each pixel, resulting in a two dimensional feature map. A sigmoid activation function is applied to each pixel of the two-dimensional feature map, providing weights in the range [0,1] for each pixel of the original feature map. The latter is called scSE block and shown in ([114], Figure 1), had the goal of combining the benefits of learning weights for spatial locations and feature channels. The two approaches work in parallel on the input feature map: a 1×1 convolution kernel is applied to obtain a two-dimensional one-channel matrix CM, while preserving the spatial dimensions. A pixel-wise sigmoid activation function is then applied to CM, finally obtaining the weight matrix, then multiplied by the input feature matrix on each channel. Two coefficients are obtained for each entry in the input feature map, and choosing their maximum value leads to best results in terms of performance and complexity added to the model [114].

EfficientNets have been introduced in [113], where the authors exploited the network scaling practice, namely, developed a novel baseline network, which can then be scaled up to obtain a more powerful model. Typically, there are 3 main dimensions, along which it is possible to scale a network: depth, width and image resolution. ResNet is a good example of the first two cases: depth ranges from basic ResNet-18 with 18 layers to architectures with 1000+ layers, while width scaling allows reaching the same accuracy as very deep ResNets with reduced training time [117]. Scaling the third dimension, image resolution, is based on the idea that better resolution of input images implies learning patterns that were not discernible at low-res; however, there is a fundamental technical limit in the memory available on the machine used for training. EfficientNets, based on MnasNet [118], implement a novel strategy—called compound scaling—for scaling base neural network architectures by depth, width and resolution, together using a set of coefficients for each dimension. Compound scaling has been validated also on common ResNet architectures and MobileNets, and can improve network performances, provided the existence of a strong baseline model. The available architectures range from the EfficientNet-b0 to the biggest EfficientNet-b7, achieving top performance on ImageNet with many fewer parameters, thus improving in efficiency. In particular, EfficientNet-b3 has $12\times 10^{6}$ parameters [113] and, tested on ImageNet for a 1000-class classification task, EfficientNet-b3 scores 81.6% in top-1 accuracy, computed as the comparison between the ground truth and the most confident prediction of the model. Furthermore, because of compound scaling, EfficientNets models support interpretability, since they focus on relevant regions when making predictions, as verified by the Class Activation Map [119]. Therefore, using EfficientNets as the encoder in a U-net architecture, allows the decoder to take advantage of the improved spatial attention mechanism of the encoder, and ultimately to improve the reconstruction of high-resolution density maps.

### 4.3. EUNet Training and Evaluation

The lymphocyte counting task was censored as a classification task, by manually defining classes of lymphocyte density. The density classes used can be represented by the set C={0,1,2,3,4,5,6}, as shown in Table 2.

Let D be a dataset represented by a collection of *n* tiles: then, $\gamma \in N^{n}$ is the vector of ground truth class for the target lymphocytes in each tile and $\hat{\gamma }\in R^{n}$ is the vector containing class predictions. As model performance metrics we used the mean absolute error (MAE), the mean-squared error (MSE), the accuracy (ACC), the Cohen’s Kappa and the Matthews correlation coefficient (MCC). MAE and MSE are the $L_{1}$ and $L_{2}$ averaged difference between predicted counts and ground truth counts, respectively, while ACC is the averaged matching between the predicted class and the ground truth class.

$$\begin{array}{cc} \mathrm{MAE} & =\frac{\sum_{i=1}^{n}|\gamma_{i}-{\hat{\gamma }}_{i}|}{n}\\ \mathrm{MSE} & =\frac{\sum_{i=1}^{n}{\left(\gamma_{i}-{\hat{\gamma }}_{i}\right)}^{2}}{n}\\ \mathrm{ACC} & =\frac{\sum_{i=1}^{n}\delta_{\gamma_{i},{\hat{\gamma }}_{i}}}{n}\;, \end{array}$$

where δ is the Kronecker delta $\delta_{\gamma_{i},{\hat{\gamma }}_{i}}=\left\{\begin{array}{c} 1\;\mathrm{if}\;\gamma_{i}={\hat{\gamma }}_{i}\\ 0\;\mathrm{otherwise}. \end{array}\right.$

The Cohen Kappa *K* [120] is a statistic measure used to evaluate agreement between two classifier, and it is defined as: $K=\frac{\mathrm{ACC}-p_{e}}{1-p_{e}}$, where $p_{e}$ is the sum of the probabilities of the two classifiers agreeing on each class by chance. *K* takes values in [−1,1] where 1 means perfect agreement between classifiers and 0 or lower values mean that the two classifier are agreeing by chance. In this work, *K* has quadratic weights for non agreeing values, thus attributing less importance to errors among nearby classes, in accordance with our classes having ordinal values.

Matthews correlation coefficient (MCC) is useful to evaluate classification performance when classes are imbalanced [121]. MCC ranges in [−1,1], where 1 and −1 mean perfect classification and complete misclassification respectively, while 0 indicates random predictions. MCC’s multiclass formula reads as follows [122,123]:

$$\mathrm{MCC}=\frac{\sum_{k,l,m=1}^{N}(C_{kk}C_{ml}-C_{lk}C_{km})}{\sqrt{\sum_{k=1}^{N}\left[\sum_{l=1}^{N}C_{lk}\sum_{f,g=1f\neq k}^{N}C_{gf}\right]}\sqrt{\sum_{k=1}^{N}\left[\sum_{l=1}^{N}C_{kl}\sum_{f,g=1f\neq k}^{N}C_{fg}\right]}}\;,$$

where $C_{a,b}$ is the number of elements in class *a* incorrectly predicted in class *b*.

The loss function used for training is the MSE between the ground truth and the predicted density map, computed pixel-wise. Since the $L_{2}$ metric penalizes large differences between pixels according to their magnitude, the larger the values of the peaks in the constructed density maps, the higher the relevance: as a result, pixels in proximity of the lymphocyte centers (where the peaks are located) are more easily predicted than pixels of lymphocyte boundaries. Coupling the Gaussian kernel density maps with the MSE loss drives the network to focus on lymphocytes centers using the context in close proximity, but without great penalty for the exact margin reconstruction.

Hyperparameter optimization is done by the Ranger algorithm [124,125], combining the Lookahead procedure [126], and the Radam stabilization strategy [127]. The rectification strategy of [127] works by tuning the variance parameters of adaptive learning rate optimizers (e.g., Adam [128]) for the first iterations, until variance stabilizes with data from a sufficient number of iterations, thus avoiding the optimizer to remain stuck in local minima. The Lookahead strategy [126] improves parameter exploration speed and stability by using two sets of weights for the optimizer. One set of weights is used for fast exploration of the loss landscape, the other set of weights updates with smaller speed and serves as a stabilizing mechanism if the state of the the optimizer get stuck in unwanted local minima of the loss function. Overall, Ranger proved to be more robust and fast with respect to Adam, warranting a stable optimization, providing a high optimal learning rate $\eta =10^{-2}$, resulting in a faster training phase, especially for the ResNet50, whose training could be reduced from more than 300 epochs to about 80 epochs.

Networks were initialized by using pretrained weights from ImageNet [110,111,112]; alternative strategies such as using weights from fine-tuned ResNet50 pretrained on the public DP dataset Lysto (https://lysto.grand-challenge.org/, accessed on 13 August 2021), did not lead to a significant performance improvement.

To guarantee robustness and reproducibility to the modeling, a preliminary training/test split with ratio 34−14 was operated and on the training set a 4×5—cross validation resampling strategy was employed, following the guidelines recommended by the US-FDS in their MAQC/SEQC initiatives [129,130]. Metrics are reported indicating average and standard deviation. Moreover, throughout the model training a particular care has been devoted into avoiding overfitting effects such as data (or information) leakage [35]: tiles extracted from the same WSI were not distributed in different training/test data subsets, a careful approach which is now becoming standard in the most recent works being published [131]. Finally, we adopted a plateau learning rate scheduler acted by monitoring metrics on validation set and reducing the learning rate if no improvements occurred for at least ten epochs: the new learning rate was computed as $\eta_{t+1}=\alpha \eta_{t}$ with α=0.2.

### 4.4. Lymphocytes Spatial Identification

The predicted lymphocytes density map is post-processed through a 3-step pipeline in order to refine the coordinates of the lymphocytes’ centers:

1. First, the predicted density map values are corrected by setting to zero all pixels with negative values. Indeed, the model learns to predict near-zero values for pixels not belonging to lymphocytes, but the prediction may tend to zero in both positive and negative direction, and for the prediction to be a valid density map the negative values should be removed.
2. Secondly, Otsu thresholding algorithm [132] is used to find an optimal value to discretize the density maps in two levels: lymphocytes and background. The Otsu algorithm is the de facto standard for discriminating foreground and background pixels within an image. In detail, the optimal threshold is identified by minimizing intra-class intensity variance (equivalent to maximizing inter-class variance). Since the Otsu algorithm is the one-dimensional discrete analog of Fisher’s discriminant analysis, this procedure coincides with globally optimizing k-means clustering on the intensity histogram. Pixels with values under the threshold are assigned to the background, while pixels with values over the threshold are assigned to the lymphocyte class.
3. Thirdly, in crowded scenarios, the simple segmentation may still result in connected components including more than one pixel. To split connected components on the Otsu mask, the Watershed segmentation algorithm [133] is used to effectively separate a dense single connected component into multiple sub-components. The result of the Watershed technique is a matrix with *n* connected components with different labels.

Finally, in order to obtain the coordinates of the center, for each connected component in the mask, the coordinates of the center of mass are computed and used as a proxy for the coordinates of the predicted lymphocytes. The goodness of the detection is evaluated by the three metrics Precision, Recall and F1-score, using as input the two sets of points *T* and *P*, defined for each tile as:

$$\begin{array}{cc} T & =\{t_{k}=\left(x_{k},y_{k}\right)|k\in \left[0,n_{1}\right],\;x_{k}\in \left[0,N-1\right],\;y_{k}\in \left[0,N-1\right]\;for\;n{}_{1}\in N,\;n{}_{1}\leq \infty \}\\ P & =\{p_{k}=\left(x_{k},y_{k}\right)|k\in \left[0,n_{2}\right],\;x_{k}\in \left[0,N-1\right],\;y_{k}\in \left[0,N-1\right]\;for\;n{}_{2}\in N,\;n{}_{2}\leq \infty \}\;, \end{array}$$

corresponding to the ground truth and the predicted center’s coordinates, respectively. The Hungarian algorithm [134] is used to find the best assignment between ground truth points and predicted lymphocyte centers. Since optimal assignment can fail if the matched points are too far away, each possible match is accepted only if the distance between points is lower than a given threshold *t*, with $\Theta \left(t\right)=s_{l}$ for $s_{l}≃4\;\mu m$, i.e., the average size of a lymphocyte [50], corresponding to 8 pixels. Accepted matches are labeled as true positive, while unmatched ground truths are considered false negatives and unmatched predictions false positives. The performance measures are defined as follows:

$$\begin{array}{cc} \mathrm{Precision} & =\frac{\left|\mathrm{True}\;\mathrm{Positives}\right|}{\left|\mathrm{True}\;\mathrm{Positives}|+|\mathrm{False}\;\mathrm{Positives}\right|}\\ \mathrm{Recall} & =\frac{\left|\mathrm{True}\;\mathrm{Positives}\right|}{\left|\mathrm{True}\;\mathrm{Positives}|+|\mathrm{False}\;\mathrm{Negatives}\right|}\\ F1 & =\frac{\mathrm{Precision}\cdot \mathrm{Recall}}{\mathrm{Precision}+\mathrm{Recall}}\;, \end{array}$$

being the F1-score the harmonic mean of precision and recall, and thus providing a unique measure to describe the overall goodness of predictions.

### 4.5. Deep Features Interpretation

To explore the hidden layers of the model, a subset $S_{N}$ of 1080 tiles was extracted from the NeSTBG dataset, 20 tiles for each available WSI, and then analysed by three different methods, namely UMAP-HDBSCAN clustering, TDA representation and TwoNN dimensionality estimation. First, UMAP is used to project data into a low *b*-dimensional space with b∈[2,12], where the upper bound is recommended in [95] for later feeding the projection into the HDBSCAN algorithm without falling into a computationally intractable task. Indeed, feature maps from raw images or from hidden layers of the network can be up to $10^{6}$ dimensions. For instance, in an intermediate step of the trained U-Net the feature map has height and width 128 and 64 feature channels, resulting in a flattened vector of length 128×128×64 = 1,048,576 elements, for each tile. Estimating densities in $10^{6}$ dimensions with approximately $10^{3}$ data points would not be feasible without the UMAP dimensionality reduction step. Value of *b* needs to be a trade-off between computational constraints (lower *b*) and the effort of preserving most of the original structure of the dataset (higher *b*): for the current tasks, b=4 was chosen. Furthermore, the UMAP minimum distance parameter was set to zero to let the embedding be free of arranging points close together; the number of neighbors parameter was set to 25 so that at each iteration UMAP is forced to compare tiles from more than one patient, since at most 20 tiles are extracted from the same WSI; finally, L2, L1 and cosine norm were used as the distance in the original feature space.

The obtained 4-dim projection was thus used as the input for HDBSCAN to extract the dense regions of the embeddings; the clustering was subsequently visualized using a different 2-dim UMAP projection for a qualitative analysis of its global structure.

Next, Betti curves are used to highlight the topological dynamics of the deep features and finally the estimate of the intrinsic dimensionality of $S_{N}$ as a point cloud is provided by the TwoNN algorithm.

Ansuini et al. [98] experimented standard convolutional neural network architectures for classification tasks (VGG, AlexNet, ResNet) and observed a characteristic pattern of intrinsic dimensionality of the deep features along layers in a well trained model. However, EUNet is more complex, with connections across multiple layers and two main branches with inherently different behaviors (encoding and decoding).

## 5. Conclusions

WSI data from DP are leveraged here to design a human-in-the-loop ML framework that could aid clinicians in NB risk assessment. As a major novelty in the pipeline, cloud computing is used to train a DL model with state-of-the-art architectures to predict density maps, an approach rarely found in DP for IHC-stained specimens. The predictive model is trained on the task of counting lymphocytes, while a post processing pipeline able to detect nuclei is implemented from the predicted density maps, with results aligned with pathologist’s estimates.

Furthermore, novel TDA approaches are employed to study the hidden representation of data as processed by the network. As future developments, different strategy for data augmentation (such as elastic transformations) or different techniques to construct the predicted target density maps can be explored, as well as possible optimization of the model architecture, and different activation and loss functions. Moreover, the current work focused on the CD3 T-cell marker as a proof of principle that can be extended to other immune cell markers to gain a deeper understanding of the role played by the immune system on NB progression.

Finally, the ML framework would strongly benefit from the ability to simultaneously recognise the tumour regions where lymphocytes are localized, e.g., septa, or tumoral nests, and to observe tiles within a larger portion of the slides, in order to gain a higher level of information.

Overall, the promising results emerging from the the current study pave the way towards the development of an effective learning tool aimed at timely and precisely quantifying the immune content within tumoral cells. Building on the awareness raising from the experience gained by previously published works [22,102,103], such a tool can work as a precious support for the pathologist, with an effective impact on the daily routine in clinical setting.

As a future development, we plan to complement the current methodological work by deepening the reported analysis through the study of the contribute of additional markers such as PD-1 and PDL-1, investigating their correlation level with both cell infiltration and patients outcome to strengthen the derived biological insights on NB.

## Figures and Tables

**Table 1:** Classification results (in terms of mean and standard deviation *mean (st.dev)* of the performance metrics for repeated experiments) for different EUNet models: in cross-validation on the training set (TR-CV), in training on the whole TR and inference on the test set, before (TS) and after (TSp) postprocessing (see Section 4.4). MCC: Matthews correlation coefficient; K: Cohen Kappa; MAE: Mean absolute error; MSE: Mean-squared error.

Subset	MCC	K	ACC	MAE	MSE
TR-CV	0.50(0.10)	0.87(0.04)	0.70(0.10)	7.0(5.0)	881(1560)
TS	0.55	0.85	0.69	3.4	47
TSp	0.59	0.84	0.71	3.1	30

**Table 2:** Lymphocyte count binning in ordinal classes.

Class	0	1	2	3	4	5	6
No. of Lymphocytes	0	1–5	6–10	11–20	21–50	51–200	>200

## Data Availability

The authors declare that all data supporting the findings of this study are available within the paper. Any other relevant data and code are available from the corresponding author upon reasonable request. The source code is written in Python/PyTorch as a deep learning framework, and it is available at the GitHub repository https://github.com/bru08/ly_decount (accessed on 13 August 2021).

## Abbreviations

The following abbreviations are used in this manuscript:

ACC	Accuracy
AI	Artificial Intelligence
ANN	Artificial Neural Network
CD3	Cluster of Differentiation 3
CNN	Convolutional Neural Networks
CT	Computer Tomography
DL	Deep Learning
DP	Digital Pathology
EUNet	U-Net with EfficientNet-b3 encoder
GB	GigaByte
HDBSCAN	Hierarchical Density-Based Spatial Clustering of Applications with Noise
IHC	Immunohistochemistry
INRGSS	International Neuroblastoma Risk Group Staging System
INSS	International Neuroblastoma Staging System
MAE	Mean Absolute Error
MB	MegaByte
MCC	Matthews Correlation Coefficient
ML	Machine Learning
MRI	Magnetic Resonance Imaging
MSE	Mean Squared Error
MYCN	v-myc avian myelocytomatosis viral oncogene neuroblastoma derived homolog
NeSTBG	Neuroblastoma Specimens with T-Lymphocytes - Bambino Gesù (dataset)
OPBG	Ospedale Pediatrico Bambin Gesù
NB	Neuroblastoma
PD	Persistent Diagram
PH	Persistent Homology
ResNet	Residual Neural Networks
RGB	Red Green Blue
scSE	spatial and channel Squeeze & Excitation (block)
SE	Squeeze & Excitation (block)
TDA	Topological Data Analysis
TIL	Tumor Inflitrating Lymphocytes
UMAP	Uniform Manifold Approximation and Projection
WSI	Whole Slide Image
